# Reconsidering the Polycystic Ovary Syndrome (PCOS)

**DOI:** 10.3390/biomedicines10071505

**Published:** 2022-06-25

**Authors:** Norbert Gleicher, Sarah Darmon, Pasquale Patrizio, David H. Barad

**Affiliations:** 1 The Center for Human Reproduction, New York, NY 10021, USA; sdarmon@thechr.com (S.D.); pxp612@med.miami.edu (P.P.); dbarad@thechr.com (D.H.B.); 2 The Foundation for Reproductive Medicine, New York, NY 10022, USA; 3 Stem Cell Biology and Molecular Embryology Laboratory, Rockefeller University, New York, NY 10065, USA; 4 Department of Obstetrics and Gynecology, Vienna University School of Medicine, 1009 Vienna, Austria; 5 Minimally Invasive Gynecology Unit, Obstetrics, Gynecology and Reproductive Sciences Department, Miller School of Medicine, University of Miami, Miami, FL 33136, USA

**Keywords:** Polycystic Ovary Syndrome (PCOS), phenotype D, androgens, on vitro fertilization (IVF), infertility, hyperactive immune system

## Abstract

Though likely the most common clinical diagnosis in reproductive medicine, the Polycystic Ovary Syndrome (PCOS) is still only poorly understood. Based on previously published research, and here newly presented supportive evidence, we propose to replace the four current phenotypes of PCOS with only two entities—a hyperandrogenic phenotype (H-PCOS) including current phenotypes A, B, and C, and a hyper-/hypoandrogenic phenotype (HH-PCOS), representing the current phenotype D under the Rotterdam criteria. Reclassifying PCOS in this way likely establishes two distinct genomic entities, H-PCOS, primarily characterized by metabolic abnormalities (i.e., metabolic syndrome) and a hyperandrogenic with advancing age becoming a hypoandrogenic phenotype (HH-PCOS), in approximately 85% characterized by a hyperactive immune system mostly due to autoimmunity and inflammation. We furthermore suggest that because of hypoandrogenism usually developing after age 35, HH-PCOS at that age becomes relatively treatment resistant to in vitro fertilization (IVF) and offer in a case-controlled study evidence that androgen supplementation overcomes this resistance. In view of highly distinct clinical presentations of H-PCOS and HH-PCOS, polygenic risk scores should be able to differentiate between these 2 PCOS phenotypes. At least one clustering analysis in the literature is supportive of this concept.

## 1. Introduction

Called “*the most common endocrine disorder of reproductive-age women*” [1,2], our understanding of the Polycystic Ovary Syndrome (PCOS) has over the last few decades stagnated. As a “syndrome”, PCOS is not a single disorder, but a basket of currently four clinical conditions called “phenotypes”, aggregated because of several common clinical denominators. Though perhaps clinically useful at the time, this classification, ultimately, may have become self-defeating [3] because of an overemphasis on clinical symptomatology, while losing sight of differences in underlying etiologies, pathophysiology, and likely, genomics.

PCOS as a syndrome arose in 1990, when the National Institutes of Health (NIH) sponsored a conference that defined PCOS by three criteria: hyperandrogenism, irregular ovulation and absence of other known fertility diagnoses [4]. In another expert conference 13 years later (Rotterdam, 2003), this definition was expanded to three diagnostic criteria: (i) oligo-amenorrhea, (ii) hyperandrogenism (clinical or biochemical), and (iii) objective evidence by ultrasound of polycystic ovaries (an ovarian PCOS phenotype, O-PCOS). A diagnosis of PCOS, however, required only two of these three clinical characteristics, thereby diluting the specificity of diagnosis, as PCOS patients now could express combinations of diagnostic criteria. This change added two phenotypes: ovulatory women with so-called O-PCOS and hyperandrogenism, and oligo-anovulatory women with O-PCOS but without hyperandrogenism, thereby defining Rotterdam criteria, to this day as phenotypes A (also called the “classical” phenotype), B, C, and D (also called the “lean” phenotype) [4].

**Figure 1:**
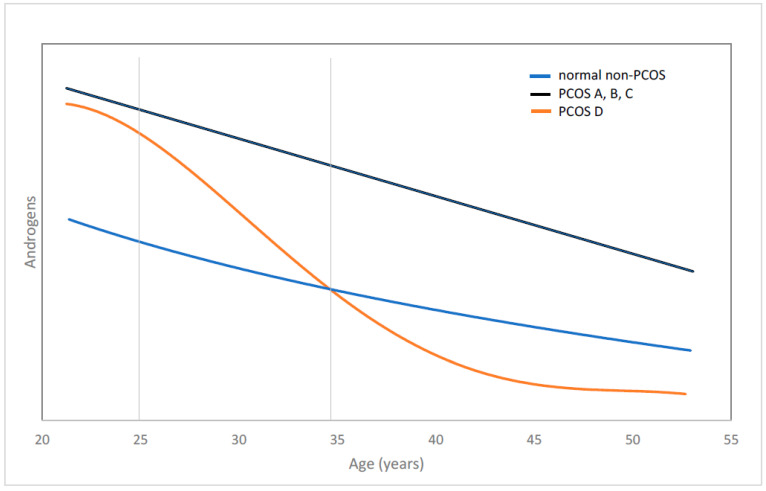
**Schematic of androgen decline with advancing age in PCOS and non-PCOS women**. The figure demonstrates androgen levels in phenotypes A, B, and C until menopause remain high in comparison to androgens in non-PCOS patients. Phenotype D, however, over approximately 10 years between ages 25–35, goes from hyper- to hypoandrogenism, at which point this phenotype becomes relatively resistant to infertility treatments, a resistance that, as shown in this manuscript, can be reversed through androgen supplementation.

The bases for our presented recommendations are observations made at our center over the last decade [5,6,7] and since have been reaffirmed through clinical management of patients (see Supporting Evidence below). The core finding of these studies was the redefinition of the “lean” phenotype D—defined under Rotterdam criteria as the “*only non-hyperandrogenic phenotype*”. Our studies established that the definition of the PCOS phenotype D as non-hyperandrogenic was incorrect. The reason for a needed correction lies in the observation that phenotype D, in contrast to the other three phenotypes, significantly changes androgen levels with advancing age (Figure 1) [6,7]. In contrast to the Rotterdam criteria, which describe only phenotypes A, B, and C as hyperandrogenic, phenotype D also starts out as hyperandrogenic between menarche and approximately age 25 [6,7]. Its precursor stage is, likely, as described by NIH investigators assessing teenagers, hyperandrogenic nodular adrenal hyperplasia [8]. The D-phenotype then is in contrast to phenotypes A, B, and C, which remain age-specific hyperandrogenic, over approximately one decade between ages of 25–35 drops androgen levels initially into normal range, only to decline further into abnormally low-levels after approximately age 35 (Figure 1) [7].

Since most PCOS diagnoses are made between the ages of 25–35 [9], when androgen levels in phenotype D PCOS patients hover in normal range, this pattern also explains why Rotterdam criteria erroneously to this day describe phenotype D as “non-hyperandrogenic.”

In almost all cases (though there are exceptions), declining androgens are caused by insufficient adrenal androgen production [6,7], responsible for approximately half of glandular androgen production, with the other half coming from ovaries. The distinction between adrenal and ovarian androgen deficiencies in association with ovarian insufficiency is important when the assumption is that this adrenal hypoandrogenism adversely affects ovarian function [10] and, as will be discussed later, is ultimately responsible for treatment resistance in phenotype D patients (here given the acronym HH-PCOS patients describing their transition from hyper- to hypoandrogenism as HH) after age 35. Hypoandrogenism of ovarian origin would, of course, indicate theca-cell insufficiency and, therefore, likely irreversible ovarian insufficiency. In contrast, adrenal hypoandrogenism, easily remedied through exogenous androgen supplementation, will “rescue” insufficient ovarian function caused by hypoandrogenism [10] (see also Supportive Evidence).

Phenotype D also differs significantly in several other aspects from the “classical” phenotype A, and phenotypes B, and C (Table 1) [6,7]. While phenotypes A, B, and C, to varying degrees, are associated with obesity, hirsutism, acne, and oligo-amenorrhea/anovulation, phenotype D usually has none of these stigmata, though as already noted, crossover phenotypes do occur. D phenotypes usually present with regular menses and have ovulatory cycles. Genomically, the most interesting difference is the close association of phenotypes A, B, and C with the metabolic syndrome, while phenotype D has almost no such association, but in approximately 85% is associated with evidence of a hyperactive immune system, mostly characterized by autoimmunity and inflammation [7]. This is one reason why the adrenal hypoandrogenism of the zona reticularis is suspected to represent an autoimmune condition. The other reason for this suspicion lies in the fact that insufficiency of the other two zonae of the adrenals is an established autoimmune disease (Addison’s disease) [6,7].

Considering these outlined differences, going forward we propose that PCOS be viewed as in principle two distinct, though at times overlapping, diagnostic entities: H-PCOS and HH-PCOS made up of two distinct conditions, the so-called (only) hyperandrogenic PCOS phenotype (H-PCOS) representing current phenotypes A, B, and C, and the hyper-/hypoandrogenic PCOS phenotype (HH-PCOS), exclusively reflecting phenotype D. It has been our experience that phenotype D in most cases, especially at older ages, goes undiagnosed and, therefore, is a more frequent infertility diagnosis than appreciated. We predict that polygenic risk scoring will be able to differentiate between H-PCOS and HH-PCOS, with H-PCOS demonstrating a genomic pattern characterized by genes important for metabolism, while the genomic pattern HH-PCOS will, likely, be characterized by immune system hyperactivity. 

### How the New PCOS Hypothesis Evolved

The European Society for Human Reproduction and Embryology (ESHRE) and the American Society for Reproductive Medicine (ASRM) in 2003 reaffirmed PCOS as “*a syndrome*”, reemphasizing that PCOS was “*not defined by any single diagnostic criterion*” [11]. Already the following year, experts, however, warned that these new criteria give rise to phenotypes that “*may not actually represent PCOS*” [12]. The same experts the following year expanded on this criticism and described the adoption of the Rotterdam criteria for definition of PCOS as “*premature*” [4].

A series of expert conferences followed [13,14,15,16]; but in many ways Rotterdam criteria to this day have remained the last word [3]. A more detailed history of PCOS phenotypes until 2016 has been presented elsewhere [17]. In this historical review, the authors pointed out two relevant observations for the presented view of PCOS: first, they noted that referral biases among PCOS patients are not uncommon, an important consideration we will return to. Second, the authors stressed the importance of age in selecting criteria for the diagnosis of PCOS. 

The effect of age on PCOS has been a subject of interest for some and, indeed, provided the initial impetus for PCOS investigations at our center [5], which ultimately led to our presented views. Recently two similar studies from different groups again addressed this subject: the first, interestingly, concluded that, “*the relative prevalence of PCOS phenotypes changes over time, menstrual irregularities normalize, androgen levels decline, as polycystic ovarian morphology improves*” [18]. More specifically, phenotype A is dominant into the 30 s, representing 50–60% of all PCOS patients before the age of 40. As noted earlier, the D phenotype in the 20s represents only ca. 15%, peaks in the 30s when it represents over 30% of all PCOS cases and shrinks again into the roughly 12–15% range in the 40s, though we have reason to believe that by this age this phenotype is often overlooked. A full 30% of PCOS patients by age 40 were no longer described as PCOS patients by these authors. We here will offer evidence that many of these seemingly no-longer PCOS patients still manifest major components of their genomic phenotype.

The argument that the distribution of phenotypes changes with advancing age must be viewed with some skepticism, because if one assumes specific genomic backgrounds for phenotypes, patients will not change phenotypes as they age, but phenotypes will change their clinical expressions in varying ways as women get older, while phenotypes, of course, genomically must remain the same.

Though also following longitudinal cohort studies of PCOS in the literature, the second paper is less revealing. Its major observation was that total testosterone and DHEA-S levels decline more significantly in PCOS than in controls patients which, of course, should not surprise since PCOS patients start from a much higher level (Figure 1). This study, moreover, offered no insight into individual phenotypes, which, unfortunately, is a major shortcoming of many PCOS studies in the medical literature [19].

With refreshing self-effacing criticism of the current status quo regarding PCOS, a recent systematic review offered further details of PCOS-related diagnostic and clinical practice guidelines [20], while yet another group of investigators in an “update on PCOS” characterized PCOS as a still “*perplexing condition*” [21]. A need for additional clarity has, thus, been recognized for some time.

Mostly representing the Androgen Excess & PCOS Society (AE-PCOS), one group of investigators has for some time held the opinion that PCOS is primarily a disorder of androgen excess. They, therefore, believe that a PCOS diagnosis should only be based on presence of hyperandrogenism in association with ovarian dysfunction. The society, consequently, excluded the presumed normoandrogenic PCOS phenotype D under the Rotterdam criteria from the diagnosis of PCOS [22,23]. Since the phenotype D plays a central role in the here proposed realignment of PCOS, these opinions, of course, have considerable relevance. To explain further, we must return to a previously noted surprising finding in 2014 that our center’s PCOS patients—almost exclusively—only were phenotype D women above age 35 [5] and that these women presented with hypoandrogenism [6,7], while less than five percent of our PCOS patient pool represented phenotypes A, B, and C.

**Table 1:** Differential diagnosis between H-PCOS and HH-PCOS phenotypes.

Characteristics.	Hyperandrogenic (H) Phenotype [15]	Hyper-/Hypoandrogenic (HH) Phenotype [5,6,23]
Appearance	Truncal obesity Hirsutism Acne	Lean BMI
Time of first clinical infertility presentation	Mostly < age 35	Mostly age > 35
Diagnosis		
Menses	Oligo-amenorrhea	Mostly ovulatory-regular
Androgens	Hyperandrogenism	Hyperandrogenism < age 25 Normal androgens at age 25–35 Hypoandrogenism > age 35
SHBG	Normal	High > age 35
LH/FSH inversion	Yes	No
AMH	High for age	High for age
FSH/AMH discrepancy	No	Yes, high AMH for FSH
DHEA/DHEA-S ratio *	~1.0	>2.0
Confirmatory findings	Family history of metabolic syndrome Metabolic syndrome O-PCOS phenotype on ultrasound	Family history of autoimmunity/inflammatory diseases Autoimmune/Inflammatory Markers Evidence of hyperactive immune system Treatment resistance to standard fertility treatments Usually milder O-PCOS phenotype on ultrasound
Past IVF experience **	Large egg numbers for age Normal egg/embryo quality	Large egg numbers for age Disproportionally few embryos Poor egg/embryo quality
Primary treatment	Ovulation induction/IVF	Androgen supplementation/IVF

* Reflective of low adrenal DHEA-S production. ** IVF, in vitro fertilization.

Considering that phenotype D under the Rotterdam criteria was supposed to be normoandrogenic and the rarest of all four PCOS phenotypes [24,25], it should not surprise that we initially were puzzled. Never before had a PCOS patient at any age been reported to be hypoandrogenic; suffice it to say, neither had a complete phenotype ever been described as becoming hypoandrogenic at any age and, as just noted, several leading PCOS authorities, indeed, have insisted that, as a basic principle, PCOS was defined by hyperandrogenism [22,23]. Refusing, in their opinion, normoandrogenic phenotype D to be included in the PCOS diagnosis, how could then a hypoandrogenic HH-PCOS above age 35 hope to be accepted?

That PCOS patients can self-select to certain fertility centers has been previously reported [17]. In our case, however, this not only meant that phenotype D patients preferably selected our center, but that phenotype A, B, and C patients at the same time deselected our center. Those two observations in combination could only be explained by the history of our fertility center. Located in New York City, the center over the last two decades progressively evolved into a fertility center of “last-resort,” catering to patients worldwide who previously had failed multiple IVF cycles at several IVF centers. Over 90% of new patients had failed prior IVF cycles elsewhere. This allowed for the conclusion that these patients obviously had not conceived and delivered after IVF cycles at other centers. It, however, also meant that patients who had conceived and delivered elsewhere, no longer had reason to become patients at our center. The only possible explanation for the highly unusual distribution of PCOS phenotypes at our center, therefore, was that PCOS patients with A, B, and C phenotypes conceived locally and therefore no-longer required the services of our center; phenotype D patients over age 35, however, for some reason failed to conceive and, therefore, had no choice but to reach out to our “last resort” center. 

This explanation further suggests that phenotype A, B, and C patients must be less IVF treatment-resistant than phenotype D patients, contradicting a longstanding perception in the infertility field that the “classical” phenotype A not only represents over half of all PCOS cases but also presents the most significant clinical challenges among all PCOS phenotypes. Accepting our offered explanation for our center’s unusual PCOS phenotype distribution, especially over the age of 35, phenotype D now, however, must be considered the more treatment-resistant and, therefore, more complex treatment target. How radical a change in perception this conclusion represents is probably best documented by the fact that a search of the English literature over the last 20 years regarding the clinical management of the D-phenotype of PCOS revealed only one single reference. In other words, phenotype D PCOS, to this day has basically been ignored in the infertility literature.

Investigating potential causes for the, now, treatment-resistant-defined phenotype D, led to the hypoandrogenism that develops in affected women by approximately age 35 [6,7]. Though still a controversial subject especially among clinicians, among biologists the importance of normal androgen levels for normal follicle maturation and growth is by now well established. [10] Elimination of hypoandrogenism through androgen supplementation, in our protocol with dehydroepiandrosterone (DHEA), apparently normalized conception rates with IVF in these patients (for further detail, see below Supporting Evidence).

Differences in observed ontogeny of PCOS phenotypes with advancing age have been reported as early as in 2013 [25]. We here suggest only *two* PCOS entities or phenotypes (Table 1): (i) An obese persistently hyperandrogenic and mostly oligo-amenorrheic, at more advanced ages also characterized by the metabolic syndrome (H-PCOS) and, (ii) an only at young ages hyperandrogenic, but after age 35 (because of progressively insufficient adrenal androgen production [6,7]), hypoandrogenic and mostly normo-ovulatory diagnostic entity, characterized by a hyperactive immune system (i.e., inflammation and autoimmunity) in ca. 85% of affected women, but without significantly increased risk for the metabolic syndrome (HH-PCOS) [6,7]. These two PCOS entities, therefore, do not only differ significantly in clinical presentation but also, likely, differ in etiologies and pathophysiology, with H-PCOS seemingly representing a primarily metabolic condition (metabolic syndrome) and HH-PCOS a more immunologically-driven condition (hyperactive immune system and, therefore, increased miscarriage risk).

The until recently incorrectly understood ontogeny of the “lean” PCOS D-phenotype, assuming absence of hyperandrogenism at all ages, prevented the recognition of HH-PCOS not only as an underdiagnosed infertility condition, but also separates this phenotype from the other three, which uniformly remain hyperandrogenic into advanced ages (Figure 1). The difference in androgen levels especially after age 35 then becomes the defining element in turning HH-PCOS into a treatment-resistant condition in comparison to H-PCOS because, as has become apparent over the last two decades, ovaries require normal peripheral androgen levels to function properly and produce good quality oocytes in sufficient numbers [10]. Since the hypoandrogenism in women with HH-PCOS almost universally is caused by insufficiency of adrenal androgen production, exogenous androgen supplementation represents effective treatment and reverses treatment resistance (see below Supporting Evidence).

The Rotterdam criteria PCOS phenotypes, therefore, basically have only two common denominators, and even those two are only shared at young ages: hyperandrogenism and abnormally high anti-Müllerian hormone (AMH) levels. High AMH values are the only truly universal finding in all PCOS patients; yet to this day they, paradoxically, are not acknowledged as a diagnostic parameter for a PCOS diagnosis [26]. This is for clinical reasons illogical; but this hormone, in addition, also offers a potential biological link between H-PCOS and HH-PCOS. With the HH-phenotype, an adrenal condition of insufficient androgen production by the zona reticularis [6,7], having been remarkably overlooked in the literature, it has been observed that, after ovaries and testes, the adrenal cortex has the by far highest concentration of AMH receptors of any human organ [27]. An adrenal AMH function has so far, however, not been described in the literature. Indeed, it could turn out to be the unifying link between H- and HH-PCOS.

In summary, our presented observations point toward a much simpler definition of PCOS than currently exists, basically returning to the concept of only two PCOS entities, H-PCOS and HH-PCOS. When clinicians currently imagine a PCOS patient, the picture they see before their eyes is usually the “classical” phenotype A patient with her typical stigmata of truncal obesity, hirsutism, and acne and a history of oligo-amenorrhea. Since the “lean” PCOS phenotype D has none of these stigmata, especially at more advanced ages, it often goes undiagnosed. Phenotype A, therefore, is likely overdiagnosed, while phenotype D, likely, is underdiagnosed [28].

It is also important to note that our review of the PCOS literature demonstrates that PCOS studies only rarely are stratified by Rotterdam phenotypes. One of the two previously noted, just recently published longitudinal studies attempting to determine the effects of age on PCOS is a good example [19]. How would anybody expect to advance the understanding of the natural history of PCOS by throwing all phenotypes into the same pot? Studies of mixed patient populations often become uninterpretable. Considering likely differences in etiology and pathophysiology, a mixed H- and HH-phenotype population would, with considerable certainty, a priori be futile.

## 2. Materials and Methods

### 2.1. Hypothesis

We noted earlier that, based on the previously noted observation that above 95% of our center’s PCOS patients are of the “lean” phenotype D, and after age 35 are described as HH-PCOS. We earlier also explained why only treatment resistance to standard fertility treatments, including IVF, potentially explains such a highly selected distribution bias among PCOS patients at our center. This assumption then allows for a secondary assumption that a successful remedy in counteracting this treatment resistance should equalize this patient population’s pregnancy chances with IVF in comparison to a non-PCOS control infertility population with equally unfavorable fertility prognosis. Assuming the reversal of hypoandrogenism through pre-IVF androgen supplementation eliminates the treatment resistance of HH-PCOS patients, so-pre-supplemented HH-PCOS patients at our centers should now demonstrate non-inferiority in IVF outcomes to matched non-HH-PCOS patients.

### 2.2. Androgen Supplementation

Because of their hypoandrogenism, HH-PCOS patients were, prior to IVF cycle start, pre-supplemented with dehydroepiandrosterone, DHEA, 25 mg TID, (Fertinatal^®^, Fertility Nutraceuticals, New York, NY, USA) for at least 6–8 weeks but usually until pregnancy. Hypoandrogenic control patients were supplemented in the same way.

### 2.3. Patient Selection

HH-PCOS patients were identified in our center’s electronic research data bank by three previously reported diagnostic criteria [6,7] [Table 1]: (i) abnormally high age-specific AMH; (ii) abnormally low age-specific androgens; and (iii) evidence for 2 out of 3 additional laboratory findings: a hyperactive immune system based on laboratory evidence of autoimmunity and/or inflammation, abnormally high sex hormone binding globulin (SHBG), which often denotes hypoandrogenism, and a DHEA/DHEA-S ratio of over 2.0, reflecting adrenal origin of a patient’s hypoandrogenism. 

Based on these three criteria, a search of our center’s electronic research data base revealed 154 HH-PCOS patients. Those patients were ultimately age- and time-matched with 126 control patients who did not demonstrate the above noted three characteristics (Table 2A). HH-PCOS patients were defined by (i) abnormally high age specific AMH for age (3rd tertial of 2186 infertile women at our center). In contrast, control patients had AMH levels in the 2nd (mid) tertial (Table 2B). (ii) By low total (TT) and/or free testosterone l (FT) levels, in HH-PCOS patients defined as TT < 20.0 ng/dL and FT < 1.2 pg/mL and in controls as TT of 20.0–33.0 ng/dL and FT as 1.2–2.4 pg/mL. Finally, as (iii), HH-PCOS patients had to demonstrate at least one of the following findings: SHBG > 80.0 nmol/mL (while controls had to be below that levels); a DHEA/DHEA-S ratio of >2.0 (while control patient had to have a ratio < 2.0) and two or more autoimmune and/or inflammatory markers from a panel of tests listed in Table 2A. Controls included couples with female and/or male infertility of varying causes but with absence of above noted HH-PCOS criteria. Controls also could not show autoimmune/inflammatory laboratory markers.

Tertials for AMH were assessed from an infertile patient population of *n* = 2186 and a control infertile patient population of 2083 women. TT and FT tertials were assessed in HH-PCOS patients from 2102 patients. Sufficiently high AMH values and low TT/FT values to satisfy a diagnosis of HH-PCOS were found in 640 women (625 controls). Adding the 3rd criterion reduced the number to 154 patients who underwent 54 fresh non-donor cycles (controls, 126 patients with 50 cycles).

### 2.4. Statistics

Continuous variables were presented as mean and standard deviation with a two-sample *t*-test and were controlled for age with a negative binomial model. Categorical variables are here presented as number and percentage, compared with Fisher’s test, and controlled for age with a logistic regression model. Statistical analyses were performed by the center’s statistician (S.D.). Data were extracted from the center’s anonymized electronic research database.

### 2.5. Informed Consent

All patients and controls had signed an informed consent that permitted the use of the patients’ medical record for research purposes, if patient anonymity was maintained, and medical records remained confidential. Since use of the electronic data base fulfilled those criteria, this research was approved by the Center for Human Reproduction Institutional Review Board (IRB) in expedited review.

## 3. Results 

Table 3 summarizes patient characteristics in HH-PCOS and control patients. As the table demonstrates, both study groups were similar in age (40.5 ± 6.0 vs. 39.3 ± 4.9 years; *p* = 0.2579). Likely, because approximately a third of newly presenting patients were already on androgen supplementation, DHEA was also similar (341.2 ± 257.2 vs. 389.6 ± 249.9 ng/dL; *p* = 0.3372). That, outside of the HH-PCOS diagnosis, both groups were matched well, was further confirmed by similar numbers of prior IVF cycles (1.2 ± 2.1 vs. 1.2 ± 2.0; *p*= 0.8435) and number of prior live births (*n* = 18, 33.3% vs. *n* = 15, 30.0%; *p* = 0.8335).

That DHEA-S (234.8 ± 191.4 vs. 175.7 ± 149.8 ug/dL; *p* = 0.0841) demonstrated only a trend toward lower levels in H-PCOS patients was likely also due to many patients already being on DHEA supplementation when presenting to our center. That HH-PCOS patients in the study, however, represented well previously outlined characteristics of the HH-PCOS diagnosis, was demonstrated by a significantly higher DHEA/DHEA-S ratio in HH-PCOS patients (2.6 ± 1.0 vs. 1.6 ± 1.9 ± 0.8; *p* = 0.0128) and significantly higher AMH values in HH-PCOS (2.0 ± 1.5 vs. 0.7 ± 0.7 ng/mL; *p* < 0.0001), confirming their higher FOR. While total testosterone (TT) did not differ between the two groups (22.0 ± 13.8 vs. 24.4 ± 3.3 ng/dL; *p* = 0.2508), free T (FT) was significantly lower in HH-PCOS patients (1.1 ± 1.0 pg/mL vs. 1.7 ± 0.3 pg/mL); *p* = 0.0003) and SHBG was significantly higher (115.1 ± 51.1 vs. 63.5 ± 33.6 nmol/mL, *p* < 0.0001), further supporting the observation of lower T in women with HH-PCOS. Finally, HH-PCOS patients also demonstrated significantly more laboratory findings suggestive of a hyperactive immune system (2.6 ± 0.9 vs. 1.6 ± 0.8; *p* = 0.0481).

Table 4 summarizes IVF cycle outcomes: clinical pregnancy rates at our center were assessed in only first IVF cycles and, again, cumulatively in all the cycles patients underwent. No outcome differences were apparent in either analysis. Assessing only first cycles, cancellations did not differ (8, 14.8%) vs. 9 (18.0%; *p*= 0.7922, adjusted for age, *p* = 0.6392), neither did numbers of oocytes retrieved (5.9 ± 6.0 vs. 7.8 ± 0.1; *p* = 0.1990, adjusted for age, *p* = 0.0950) or number of embryos transferred (1.4 ± 1.3; *p* = 0.4892, adjusted for age, *p* = 0.4892). If cumulative pregnancy and live birth rates were analyzed, also no differences in outcomes were apparent (*p* = 0.8253). At least 1 clinical pregnancy was achieved in 12 women in both groups (22.2% and 24.0%; *p* = 1.0000) and live births in 11 (20.4%) vs. 8 (16%, 0%; *p* = 0.6187). HH-PCOS patients, thus, demonstrated no inferiority in IVF outcomes in comparison to controls. Outcomes, indeed, were remarkably similar.

## 4. Discussion

Here, our offered evidence supports the contention that a well-defined homogenous group of HH-PCOS patients (phenotype D over age 35), if properly pre-supplemented with androgens (in this case DHEA), no longer demonstrated the presumed treatment resistance to IVF that brought them to our center in the first place. Since their outcomes exactly matched those of non-HH-PCOS patients, the conclusion that hypoandrogenism is the underlying cause of their treatment resistance to IVF appears indisputable; that is, if one accepts the hypothesis that our center’s highly biased PCOS distribution, reflected in phenotype D representing over 95% of all PCOS patients, is the result of prior treatment resistance.

We furthermore conclude the following: (i) As increasingly evident, [18,19,25] going forward, PCOS diagnoses must be age specific. (ii) Up to approximately age 25, all PCOS women share only two characteristics: hyperandrogenism and high AMH values. After age 25, androgen levels are no longer defining for all PCOS phenotypes and other criteria must be relied on in addition [6,7]. (iii) At all ages the only uniformly present diagnostic tool for PCOS is abnormally high age-specific AMH. Though experts have suggested otherwise [26], AMH, paradoxically, is, nevertheless, still *not* formally considered a diagnostic test for PCOS. (iv) PCOS represents only two distinct entities, an at all ages persistently hyperandrogenic (H-PCOS), encompassing Rotterdam criteria phenotypes A, B, and C, and an initially hyper-, but after age 35 hypoandrogenic (HH-PCOS) phenotype, mostly representing the lean phenotype D. (v) Both presentations are, however, most distinguished by their obvious genomic differences; one, a primarily metabolic condition (H-PCOS), and the other, mostly an immunologic/inflammatory condition, with the latter entity’s hypoandrogenism almost always exclusively adrenal in origin and, likely, mostly of autoimmune etiology [6,7].

A question that arises is, whether these two conditions are phenotypes of one syndrome or, because of their obvious differences in genomics, etiology, and pathophysiology, should ultimately not be viewed as two distinct medical conditions? A recent unsupervised phenotypic genetic clustering study of women with PCOS, indeed, offers strong support for the latter conclusion and, therefore, also for here proposed restructuring of how PCOS should be viewed: [29] The authors of this study revealed two “distinct” PCOS subtypes in full agreement with the here proposed two entities. A first, by the authors called the “reproductive” group (21% to 23% of their two young study populations at median age 28), characterized by higher SHBG and lower BMI as well as insulin levels, therefore, compatible with here proposed HH-PCOS population, and a second “metabolic” group (37–39% of patients), characterized by higher BMI, glucose, and insulin levels but lower SHBG and, therefore, fully compatible with the here proposed H-PCOS entity. The investigators, moreover, found that, as our proposal hypothesizes, their two newly described entities were associated with novel susceptible loci.

In addition, noteworthy is that already in 2007, Kurzrock and Cohen proposed a likely male counterpart to H-PCOS, upstream caused by a genetic endocrine/metabolic susceptibility that men and women share; in men characterized by excessive hairiness and early onset of male-pattern alopecia [30]. More recently, this concept was reaffirmed by other investigators [31]. Because of such genetic similarities in men and women, an abstract at ENDO 2021 concluded that PCOS (in our interpretation H-PCOS) should be viewed more as a cardiometabolic condition than a disorder of female reproductive function, and genetic risk factors should be defined to understand causes and develop specific treatments [32]. The suggestion that H-PCOS may have a counterpart in males not only supports the possibility of a distinct genomic background for this diagnosis in both sexes but also offers the likelihood that HH-PCOS may also have a male counterpart, likely similarly characterized by early adrenal hypoandrogenism. With advancing age, hypoandrogenism in males is, of course, a very common medical finding [33].

## 5. Conclusions and Limitation

A close association of H-PCOS with the metabolic syndrome is well established, while HH-PCOS apparently has no such association [28]. In contrast, the HH-phenotype in approximately 85% of cases demonstrates evidence of a hyperactive immune system, mostly characterized by autoimmunity and inflammation [6,7]. Chronic low-grade inflammation has been universally associated with PCOS [34], but one must wonder whether this generalization is not, once again, only based on patient selection biases from mixed studies of phenotype A and phenotype D PCOS patients. This interpretation is supported by the fact that immune system hyperactivity in the HH-PCOS significantly exceeds what has been reported for PCOS in general [6,7].

Metabolic syndrome and blatantly hyperactive immune systems represent distinctively different genetic traits and, therefore, must clearly receive further research attention in carefully defined study populations. In absence of accurate differentiation between these two conditions, further progress in the understanding of PCOS will be difficult to achieve.

The principal limitation of this study lies in the assumption that our center’s highly unusual distribution of PCOS patients based on Rotterdam criteria can only be explained by patient self-selection. If, as we concluded, there is no other explanation why our center in many years hardly has seen phenotype A, B, and C PCOS patients, but has been flooded with phenotype D females, one also has to accept that this self-selection to our center is based on treatment failures elsewhere that phenotype A, B, and C patients did not experience. Increased treatment failures, of course, point toward increased treatment resistance of phenotype D patients, which as here reported case-control study demonstrated, is remedied by androgen supplementation.

This study, thus, offers a potentially new understanding of PCOS, suggests in HH-PCOS an underdiagnosed medical entity especially in women above age 35 and, in addition, offers a simple treatment option for these patients through androgen supplementation.

## Figures and Tables

**Table 2:** (**A**): **Selection criteria for HH-PCOS and control patients**. * Because the hypoandrogenism is of adrenal origin, DHEAS is usually significantly lower than DHEA. For patient selection in this study, at least a ration of 2.0 was required to qualify as a HH-PCOS patient. Except for the LA, which is expected to be negative, all other immune tests have normal ranges. A result was considered positive if this normal range was exceeded. (**B**): **Age-specific AMH cut-offs** defining upper and lower tertial, with 2nd tertial in between.

**A**				
DIAGNOSTIC CRITERIA FOR HH-PCOS		HH-PCOS *n* = 54		Controls *n* = 50
1. AMH (ng/mL)		3rd tertial		2nd tertial
2. Testosterone (T)				
TT (ng/dL)		<20.0 (1st tertial)		20.0–33.0 (2nd tertial)
FT (pg/mL)		<1.2 (1st tertial)		1.2–2.38 (2nd tertial)
3. AT LEAST 2/3 OF FOLLOWING ADDITIONAL MARKERS				
SHBG (nmol/L)		>80.0		<80.0
DHEA/DHEAS RATIO *		>2.0		0.5–2.0
>2 IMMUNE and/or INFLAMMATORY MARKERS		any positives in: Lupus anticoagulant (LA) and antiphospholipid antibodies +TPO Abs, +TG Abs, CRP, IL-6, any gammopathy in IgG, IgM, IgA, IgE		
**B**				
	Age-specific AMH (ng/mL)			
Age	Upper tertial cutoff		Lower tertial cutoff	
30	3.49		1.05	
31	3.50		1.40	
32	2.55		0.91	
33	2.60		0.80	
34	2.18		0.70	
35	1.80		0.36	
36	1.60		0.38	
37	1.30		0.32	
38	1.27		0.30	
39	0.90		0.20	
40	1.07		0.30	
41	0.96		0.21	
42	0.83		0.16	
43	0.72		0.19	
44	0.63		0.16	
45	0.50		0.16	

**Table 3:** HH-PCOS and control non-PCOS patient characteristics.

Patient Characteristics	H-PCOS Patients (*n* = 54)	Control Patients (*n* = 50)	*p*-Value
Age (years)	39.4 ± 4.9	40.5 ± 6.0	0.2579
Prior IVF cycles elsewhere	1.2 ± 2.1	1.2 ± 2.0	0.8435
Prior live births (*n*/%)	18 (33.3)	15 (30.0)	0.8335
DHEA (ng/dL)	389.6 ± 249.9	341.2 ± 257.2	0.3370
DHEAS (ug/dL)	175.7 ± 149.8	234.8 ± 191.4	0. 0841
DHEA/DHEAS ratio	2.6 ± 1.0	1.9 ± 1.6	0.0128
AMH (ng/mL)	2.0 ± 1.5	0.7 ± 0.7	<0.0001
TT (ng/dL)	22.0 ± 13.8	24.4 ± 3.3	0.2508
FT (pg/mL)	1.1 ± 1.0	1.7 ± 0.3	0. 0003
SHBG (nmol/mL)	115.1 ± 51.1	63.5 ± 33.6	<0.0001
Immune/inflammatory markers	2.0 ± 0.9	1.6 ± 0.8	0.0481

This table demonstrated how well HH-PCOS and control non-PCOS patients were matched. Significant differences only noted were related to definition of patient group: HH-PCOS patients had significantly higher AMH, significantly lower free testosterone (FT), significantly higher SHBG and significantly more inflammatory/immune markers.

**Table 4:** IVF cycle outcomes.

	H-PCOS Cycles (*n* = 54)	Control Cycles (*n* = 50)	*p*-Value/Adjusted for Age
First IVF Cycles at Center			
Cycle cancellations (*n*/%)	8 (14.8)	9 (18.0)	0.7922/0.6392
Oocytes retrieved (*n*)	5.9 ± 6.0	7.8 ± 9.1	0.1990/0.0950
Embryos transferred (*n*)	1.4 ± 1.3	1.6 ± 1.6	0.4892/0.5779
Clinical pregnancies * (*n*/%)	8 (14.8)	8 (16.0)	1.0000/0.7349
Live births (*n*/%)	8 (14.8)	5 (10.0)	0.5591/0.4863
Cumulative Ivf Cycles at Center			
Number of cycles/patient (*n*/%)			0.8253
*n* = 1	24 (44.4)	27 (54.0)	
*n* = 2	13 (24.1)	10 (20.0)	
*n* = 3	7 (13.0)	5 (10.0)	
*n* = 4+	10 (18.5)	8 (16.0)	
At least 1 clinical pregnancy (*n*/%)	12 (22.2)	12 (24.0)	1.0000
At least 1 live birth (*n*/%)	11 (20.4)	8 (16.0)	0.6187

* Pregnancy with fetal heart, visualized on ultrasound.

## Data Availability

Data supporting here reported results are available in the research data depository of the CHR by contacting J. Tapper (jtapper@thechr.com) or the CHR’s statistician and co-author, S. Darmon (sdarmon@thechr.com).
